# A returning migrant worker with avian influenza A (H7N9) virus infection in Guizhou, China: a case report

**DOI:** 10.1186/s13256-015-0580-1

**Published:** 2015-05-12

**Authors:** Dingming Wang, Guangpeng Tang, Yan Huang, Chun Yu, Shijun Li, Li Zhuang, Lin Fu, Shiping Wang, Nanshi Li, Xiyan Li, Lei Yang, Yu Lan, Tian Bai, Yuelong Shu

**Affiliations:** 1 Guizhou Provincial Center for Disease Control and Prevention, 101 Bageyan Road, Guiyang City, 550004 Guizhou Province China; 2 Zunyi Prefecture Center for Disease Control and Prevention, 19 Xima Road, Zunyi City, 563002 Guizhou Province China; 3 Meitan County Center for Disease Control and Prevention, 99-8 Zhedanan Road, Meitan County, 564100 Guizhou Province China; 4 National Institute for Viral Disease Control and Prevention, Chinese Center for Disease Control and Prevention, Key Laboratory for Medical Virology, National Health and Family Planning Commission, 155 Changbai Road, Beijing, 102206 P. R China

**Keywords:** H7N9 virus, Influenza, Migrant worker, Imported case, Epidemiology investigation

## Abstract

**Introduction:**

Human infection with avian influenza A (H7N9) virus was first reported on March, 2013 in the Yangtze River Delta region of China. The majority of human cases were detected in mainland China; other regions out of mainland China reported imported human cases, including Hong Kong SAR, Taiwan (the Republic of China) and Malaysia, due to human transportation. Here, we report the first human case of H7N9 infection imported into Guizhou Province during the Spring Festival travel season in January 2014.

**Case presentation:**

In early January 2014, a 38-year-old healthy Chinese man, a migrant worker returning from previously H7N9-affected Zhejiang Province, was identified as the first human case of infection with avian influenza A(H7N9) virus in Guizhou Province. He developed fever in Zhejiang at the beginning of January 2014, and returned to Guizhou for the Chinese New Year the next day. He went to seek medical care, but deteriorated rapidly and died on day 8 after his illness onset. The influenza virus A/Guizhou/01502/2014 isolated from the patient had 99% identity with viruses circulating in the Yangtze River Delta region. Selected amino acids substitutions, well-known to be associated with mammalian adaptation, viral replication and drug resistance were similar to other H7N9 viruses circulating in humans.

**Conclusions:**

Epidemiology investigation and laboratory results confirmed it was the first imported case of H7N9 infection in Guizhou Province. This finding further indicated that more human H7N9 cases may be detected in other regions due to frequent travel both domestically and internationally.

## Introduction

The first wave of human infections with avian influenza A(H7N9) virus began in spring and subsided by summer 2013 in China [1], and it has re-emerged since October 2013 [2]. As of March 10, 2014, a total of 379 cases and 135 deaths were reported in mainland China [3]. However, human cases were also detected in Hong Kong SAR, Taiwan (the Republic of China) [4] and recently the Malaysian Ministry of Health announced the first imported case outside of China on February 12, 2014 [5]. It indicated that human cases could be exported to other regions by frequent travel both domestically and internationally. Each year, billions of people migrate for the Spring Festival in China, therefore increasing the risk of infectious diseases spreading. Here we report the first human case of H7N9 infection imported to Guizhou Province from the epidemic region during the period of the so-called largest annual human migration.

## Case presentation

**Figure 1:**
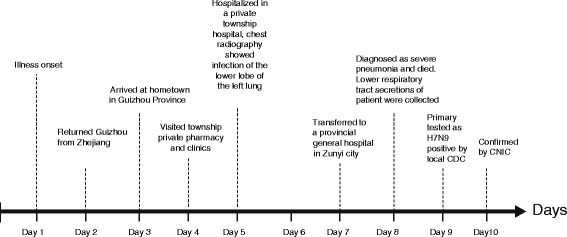
Timeline of potential exposure and medical consultation of a laboratory-confirmed imported influenza A(H7N9) case, Guizhou Province, China, January 2014.

The patient was a 38-year-old healthy Chinese man, a migrant worker who worked in Zhejiang Province. As shown in Figure 1, he developed influenza-like illness (ILI) symptoms, including headache and 40°C fever at the beginning of January 2014, and sought medical attention in a village clinic and received infusion therapy. He went back to his hometown in Guizhou Province for the coming Spring Festival by taking a long-distance bus from Zhejiang Province one day after he developed fever. He then sought medical care in a village clinic and a township hospital when he arrived at his hometown. His chest radiograph showed pneumonia in the lower lobe of his left lung (the CT value ranged from −12 to approximately −19) and he was hospitalized in a township hospital on day 5 after his illness onset. He experienced high fever and rapid progression, and was transferred to a provincial general hospital in Zunyi city. He was diagnosed as severe pneumonia, deteriorated rapidly and died on day 8 after his illness onset. He had received symptomatic treatment during the clinical treatment and all drugs that had been used in the township hospital and the general hospital in Zunyi city are listed in Table 1. No antiviral drugs were administrated during the clinical treatment. The case was reported through the Chinese surveillance system for pneumonia with unknown etiology and confirmed as human infection with avian influenza A(H7N9) virus by means of RT-PCR, virus isolation and full genome sequencing at the Chinese National Influenza Center (CNIC). Since the patient had died, written informed consent was obtained from the patient’s next-of-kin for publication of this case report.

**Table 1:** **Drugs used during clinical treatment in hospital**

**Days after illness onset**	**Treatment**
Day 5	Antibiotic treatment with vancomycin+ceftazidime+aminoglycoside
	Use of omeprazole to prevent stress ulcer
	Use of dexamethasone to reduce inflammatory exudation
Day 7	Meropenem
	Compound aminopyrine and acetaminophen
	Ceftriaxone, bromhexine, doxofylline and coenzyme complex

Sixty-one close contacts were identified by epidemiological survey, including 36 health-care personnel, eight medical escorts and 17 households and family contacts. As a public health response, written informed consents from contacts can be waived according to Chinese law. The close contacts were medically monitored for seven consecutive days; no ILI symptoms were observed in all contacts except for the patient’s family members. One family member appeared with a sore throat and developed a 38°C fever five to seven days after the patient’s illness onset, and another developed a sore throat without fever seven days after the patient’s illness onset. Continuous throat swabs were collected from them; all the samples were negative for H7N9 influenza virus. Antibody response to H7N9 virus was not detected from the paired sera of all contacts.

**Figure 2:**
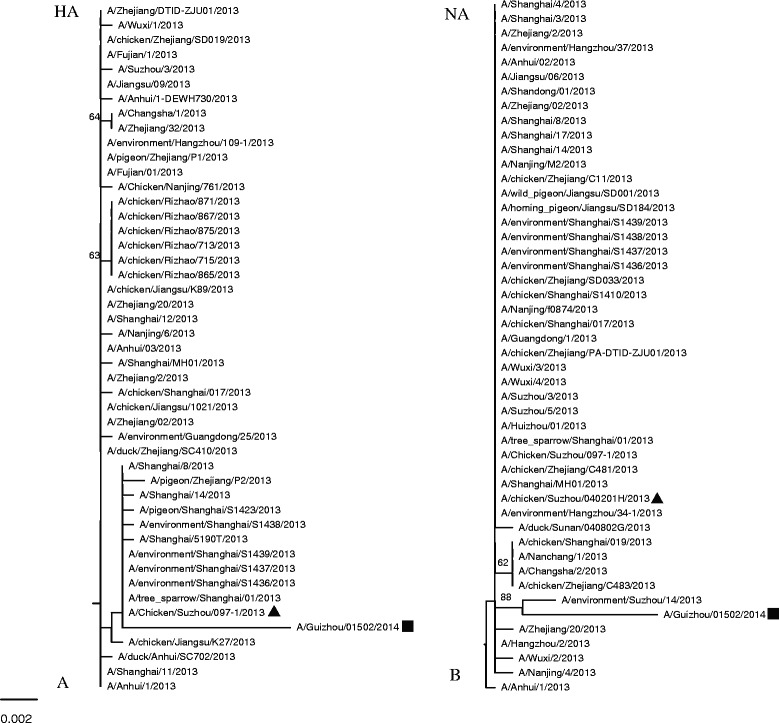
Phylogenetic tree of HA and NA gene segments of the Guizhou isolate. The H7N9 viruses that were most like the Guizhou isolate were analyzed for the two surface genes, HA **(A)** and NA **(B)**. The Guizhou isolate was marked with a black square and the most likely virus was marked with a black triangle. The bootstrap values ≥0.6 are shown at the major nodes of the phylogenetic trees.

**Figure 3:**
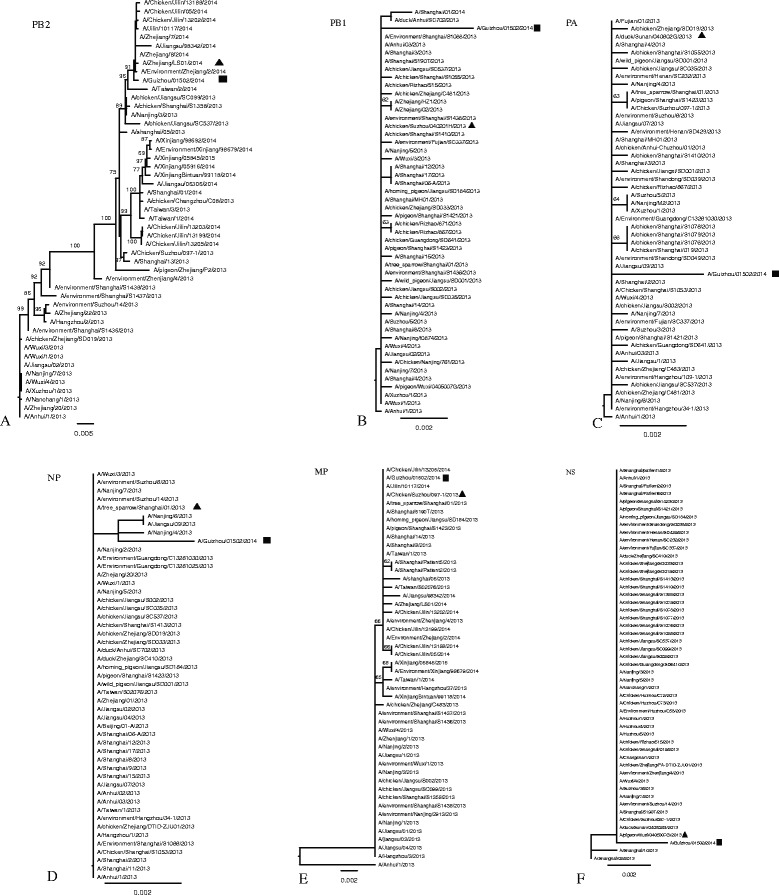
Phylogenetic tree of six internal gene segments of the Guizhou isolate. The H7N9 viruses that were most like the Guizhou isolate were analyzed for six internal genes, PB2 **(A)**, PB1 **(B)**, PA **(C)**, NP **(D)**, MP **(E)**, NS **(F)**. The Guizhou isolate was marked with a black square and the most likely virus was marked with a black triangle. The bootstrap values ≥0.6 are shown at the major nodes of the phylogenetic trees.

The influenza virus A/Guizhou/01502/2014 was isolated from the patient. The PB2 gene had a high degree of similarity (99%) to viruses isolated from human in Zhejiang Province. Another seven segments showed the highest identity with viruses isolated from birds in the Yangtze River Delta region, where Zhejiang Province is located (Table 2). The phylogenetic analysis further demonstrated all eight gene segments were clustered with viruses that circulated in this region (Figures 2 and 3). A/Shanghai/1/2013 (SH1, the first H7N9 virus isolate), A/Anhui/1/2013 (AH1, the vaccine strain recommended by the World Health Organization) and those six virus isolates with highest identity with A/Guizhou/01502/2014 mentioned in Table 2 were selected for characteristic comparison of the critical amino acids residues (Table 3). Similar to those viruses, the mammalian-adaptive mutations such as G186V and Q226L in the HA gene that were previously reported [6] were found in this virus. It remained R in the position 292 of the NA gene and suggested the effectiveness of oseltamivir antiviral treatment. The virus possessed a 627K mutation in the PB2 gene, which was proposed to be associated with high polymerase activity and increased the virulence in mice [7]. The PB1 L368V mutation was found in this virus, which increased transmission in ferret [8]. The signature amino acids of the human influenza virus in the PA gene were detected, including V100A, K356R and S409N [9,10]; however, a unique mutation L336M in the PA gene that had only been reported in a Hong Kong isolate [11] was not detected.

**Table 2:** **The highest homology of the virus isolated from the patient with other H7N9 viruses**

**Segment**	**Strain**	**Identity**
PB2	A/Zhejiang/LS01/2014 (H7N9)	2272/2280 (99%)
PB1	A/chicken/Suzhou/040201H/2013 (H7N9)	2265/2274 (99%)
PA	A/duck/Sunan/040802G/2013 (H7N9)	2145/2151 (99%)
HA	A/chicken/Suzhou/097-1/2013 (H7N9)	1668/1683 (99%)
NP	A/tree_sparrow/Shanghai/01/2013 (H7N9)	1493/1497 (99%)
NA	A/chicken/Suzhou/040201H/2013 (H7N9)	1389/1398 (99%)
MP	A/chicken/Suzhou/097-1/2013 (H7N9)	981/982 (99%)
NS	A/pigeon/Wuxi/0405007G/2013 (H7N9)	836/838 (99%)

**Table 3:** **Comparison of selected molecular markers between A/Guizhou/01502/2014 and representative H7N9 viruses**

**Protein**	**Phenotypic consequences** ^**a**^	**Amino acid position/motif** ^**b**^	**GZ 01502**	**SH1**	**AH1**	**ZJLS01**	**SZ040201H**	**SN040802G**	**CK/SZ097-1**	**tree_sparrow/SH1**	**pigeon/WX/0405007G**
HA	Increased virus binding to human-type receptors (α2-6)	G186V	*V*	G	*V*	*V*	*V*	*V*	*V*	*V*	*V*
		Q226L	*L*	Q	*L*	*L*	*L*	Q	*L*	*L*	Q
NA	Enhanced virulence in mice/adaption and transmission in domestic poultry	69-73 amino acids deletion	Yes	Yes	Yes	Yes	Yes	Yes	Yes	Yes	Yes
	Related to drug resistance	R292K	R	*K*	R	*K*	R	R	R	R	R
PB2	Increased virulence in mice	E627K	*K*	*K*	*K*	*K*	E	E	E	*K*	E
	Enhanced virulence and transmission in guinea pigs	D701N	D	D	D	D	D	D	D	D	D
PB1	Increased transmission in ferret	I368V	*V*	I	*V*	*V*	*V*	*V*	*V*	*V*	*V*
	Increased replication in mammalian cells	L598P	L	M	L	L	L	L	L	L	L
PB1-F2	Increased pathogenicity in mice	87-90 amino acids in length	90	90	90	90	90	90	25	90	90
PA	Increased polymerase activity	L336M	L	L	L	L	L	L	L	L	L
	Species-associated signature positions	V100A	*A*	*A*	*A*	*A*	*A*	*A*	*A*	*A*	*A*
		K356R	*R*	*R*	*R*	*R*	*R*	*R*	*R*	*R*	*R*
		S409N	*N*	*N*	*N*	*N*	*N*	*N*	*N*	*N*	*N*

^a^The mutation to the right of the amino acid position confers the phenotype described below.

^b^The HA gene was under the H3 numbering system. The NA gene was under the N2 numbering system. Other internal genes were numbered from the start codon (Met).

All letters in italics represent mutations detected at the relative position.

## Conclusions

This is the first imported human H7N9 case of a migrant worker moving from epidemic areas to unaffected regions during the Spring Festival migration, which is a period of travel in China with an extremely high traffic load around the time of the Chinese New Year.

Unlike most previously reported fatal cases of patients who were older and/or who had underlying medical conditions [12], this patient was young and reported without any underlying medical conditions. No mutations associated with antiviral resistance were detected; one possibility for the rapid deterioration and death of the patient may be due to the fact antiviral drugs were not used during his treatment. The virus isolated from the patient showed a high identity with viruses circulating in the Yangtze River Delta region and he developed respiratory syndromes before his arrival in Guizhou Province. He visited a neighborhood live poultry market before his illness onset, which indicated the potential infection source was likely to be the infected poultry in the market, as previously reported elsewhere [12-14]. Further, the H7N9 virus was not detected in Guizhou based on the results of poultry-related environmental surveillance since 2009 (data not shown). These data concluded that the case had been introduced from an affected region by the movement of people. Imported human cases of the H7N9 virus travelling from affected areas to non-endemic areas domestically and internationally have been reported in Hong Kong SAR, Taiwan (the Republic of China) and Malaysia during the first and second wave of H7N9 infections [4,5,15]. In each year, millions of migrant workers from rural areas of Guizhou Province go to work in the Yangtze River Delta and the Pearl River Delta and return home for a reunion with their family. With the increasingly frequent movement of people, it is foreseen that more human H7N9 cases may be detected in other regions due to frequent travel both domestically and internationally. Considering the rapidly progressive pneumonia, acute respiratory distress syndrome (ARDS), the high rates of ICU admission and death, early detection and antiviral drug therapy of suspected cases imported from affected areas are essential to reduce the case fatality rate of H7N9 virus infection.

The undetected H7N9 virus of the respiratory specimens and the negative antibody response to H7N9 in his family members who presented clinical syndromes indicated that the transmission from the patient to his family members could be ruled out.

## Consent

Written informed consent was obtained from the patient’s next-of-kin for publication of this case report and any accompanying images. A copy of the written consent is available for review by the Editor-in-Chief of this journal.
